# Immune checkpoint inhibitor-related myotoxicity in lung cancer: a decade-long real-world pharmacovigilance analysis of the FAERS database

**DOI:** 10.3389/fphar.2026.1875873

**Published:** 2026-09-22

**Authors:** Cuiping Liu, Su Wei, Xu Qi

**Affiliations:** Department of Respiratory and Critical Care Medicine, The First Affiliated Hospital of Nanjing Medical University, Nanjing, China

**Keywords:** FAERS database, immune checkpoint inhibitors, myotoxicity, pharmacovigilance, triple M overlap syndrome

## Abstract

**Background:**

Immune checkpoint inhibitor therapy has revolutionized the treatment of several malignancies, with improved survival, yet the accompanying myotoxicity has often been underestimated. We aimed to comprehensively and comparatively analyze the risk of myotoxicity associated with various ICIs using the FDA Adverse Event Reporting System database.

**Methods:**

Data spanning from the first quarter of 2015 to the fourth quarter of 2024 were extracted from the U.S. Food and Drug Administration Adverse Event Reporting System database. Disproportionality analysis was conducted using the reporting odds ratios (RORs) and corresponding 95% confidence intervals (CI) to identify potential safety signals associated with ICI-related myotoxicity.

**Results:**

From 2015 to 2024, a total of 45,266 ICI-related myotoxicity reports were reported, of which 1,515 met the inclusion criteria, with a higher proportion of male patients (n = 904,59.7%). The median age was 69.4 years (interquartile range 49.9–77.6), and 46.2% of the patients were aged 65–85 years. A total of eight positive signals were identified, mainly myalgia [N = 366, reporting odds ratio = 1.25,95%confidence intervals (1.1–1.41), proportional reporting ratio = 1.25, χ2 = 12.55] and muscular weakness [N = 317, ROR = 1.53,95%CI (1.33–1.75), PRR = 1.53, χ2 = 37.75]. Among these events, polymyositis and rhabdomyolysis showed the highest proportions of fatal outcomes (42.86% and 38.83%, respectively). Among myositis-related reports, Triple M overlap syndrome was identified in 5.6% of cases, with fatal outcomes reported in 46.75% of these reports. In terms of onset time, 49.6% of the cases occurred within the first month of treatment (n = 303). The onset time of atezolizumab was the earliest (median 21 days), while durvalumab and Pembrolizumab showed longer or delayed risk windows (median 36 days and 34 days, respectively). And there were significant differences in the onset time of different ICI-related myotoxicity (P = 0.003).

**Conclusion:**

This FAERS-based pharmacovigilance study identified heterogeneous reporting signals and clinical patterns of ICI-associated myotoxicity. Most reported events occurred within the first 2 months of treatment, while myositis overlap syndromes, particularly the Triple M phenotype, were associated with higher proportions of fatal or life-threatening outcomes. These findings highlight the importance of early recognition and multidisciplinary monitoring of ICI-associated myotoxicity. Further clinical studies are warranted to validate these findings and clarify their underlying mechanisms.

## 1 Introduction

Despite advances in treatment, lung cancer remains the leading contributor to cancer mortality worldwide (Li M. et al., 2024). The rapid development of immunotherapy, especially the progress of immune checkpoint inhibitors (ICIs), re-activates the inhibited T cell response by blocking the PD-1/PD-L1 and CTLA-4 axes, and changes the treatment mode of advanced non-small cell lung cancer (Pardoll, 2012; Kinoshita et al., 2021; Ghemrawi et al., 2024). Although ICIs such as pembrolizumab and nivolumab provide significant therapeutic survival benefits, they also cause a series of immune-related side effects (irAEs), which are significantly different from the toxic reactions caused by traditional chemotherapy (Liu et al., 2020). With the promotion of clinical applications, it has become an urgent clinical priority to describe real-world safety profiles and identify severe toxicity reporting patterns.

The therapeutic mechanism of immunotherapy inevitably disrupts immune homeostasis and compromises self-tolerance (Li et al., 2015; Knauss et al., 2019). Such off-target effects can trigger the activation of self-reactive T cells and inflammatory responses in healthy tissues, resulting in a wide spectrum of irAEs (Ramos-Casals et al., 2019; Fang et al., 2022). With the increasing use of ICIs, the documented incidence of irAEs has surged, spanning nearly all organ systems and ranging from mild functional impairment to life-threatening complications that necessitate permanent treatment cessation (Hu et al., 2025; Zhang et al., 2025). A global pharmacovigilance analysis using VigiBase, an international database collecting spontaneous safety reports from more than 130 countries, found that the proportion of reported irAEs classified as ICI-associated myotoxicities increased significantly from 3.7% before 2017 to 8.3% in 2021–2022 (Gougis et al., 2024). Although myotoxicity still represents a relatively small proportion overall, ranking seventh among documented irAEs subtypes, its rapid emergence marks it as an increasingly prominent clinical challenge in the modern immuno-oncology era (Salem et al., 2026). The insidious onset and nonspecific early symptoms of myotoxicity often lead to diagnostic delays, potentially resulting in profound morbidity or fatal outcomes. Consequently, timely recognition and evidence-based management of ICI-related myotoxicity are paramount to safeguarding patient safety and optimizing the survival benefits of immunotherapy (Angelopoulou et al., 2021).

Current research on ICI-induced myotoxicity is limited by insufficient sample sizes and short follow-up durations in clinical trials. As a result, ICI-related myotoxicity may be underreported or overlooked, hindering a comprehensive assessment of the long-term safety of ICIs. The FDA Adverse Event Reporting System (FAERS) is a global spontaneous reporting database that collects adverse events associated with approved drugs, and provides real-world post-marketing safety information that complements clinical findings. Our study aims to leverage the FAERS database to characterize reports of ICI-related myotoxicity, with a particular focus on lung cancer—one of the most common malignancies associated with irAEs (Das and Johnson, 2019), and to provide insights for clinical monitoring and management.

## 2 Materials and methods

### 2.1 Data source and collection

FAERS is a publicly accessible repository that collects voluntary reports of adverse drug events (AEs) from healthcare professionals, drug manufacturers, and patients. In this study, FAERS data were used to retrieve reports of muscle toxicity associated with immune checkpoint inhibitors among patients with lung cancer between the first quarter of 2015 and the fourth quarter of 2024. This work was designed as a retrospective pharmacovigilance/disproportionality analysis rather than a case-control study.

The generic drug and brand name were used to identify AE cases associated with ICI agents. The following ICIs were included in the analysis: Nivolumab (Opdivo), Pembrolizumab (Keytruda), Atezolizumab (Tecentriq) and Durvalumab (Imfinzi). Drugs were categorized as primary suspect, secondary suspect, concomitant, or interacting. Primary suspect cases were defined as reports in which a target ICI was coded as the primary suspect drug. Only these reports were included in the primary analysis. Patient demographic information (e.g., age, sex, reporter type), clinical data (e.g., indication, outcome), and the time to onset of adverse events (TTO) were also recorded. TTO was defined as the interval between the date of treatment initiation (START_DT in the THER file) and the occurrence date of the adverse event (EVENT_DT in the DEMO file). To ensure data quality, duplicate reports were removed according to the FDA-recommended procedure using three identifiers: the case identifier (CASEID), FDA receipt date (FDA_DT), and primary report identifier (PRIMARYID). Specifically, when multiple reports had the same CASEID, only the report with the most recent FDA_DT was retained. If both CASEID and FDA_DT were identical, the report with the highest PRIMARYID was retained. Data processing and visualization were performed using Microsoft Excel 2021 and R software (version 4.4.3). The core principle of this customized approach is to maximize specificity and sensitivity.

All the AEs were coding using the Medical Dictionary for Regulatory Activities (MedDRA; version 27.1). MedDRA has a five-level hierarchical structure consisting of Lowest Level Term (LLT), Preferred Term (PT), High-Level Term (HLT), High-Level Group Term (HLGT), and System Organ Class (SOC). In this study, adverse events were identified at the PT level and categorized according to their corresponding SOC. Myotoxicity cases were identified using a customized list of Preferred Terms derived primarily from the Standardized MedDRA Query “Rhabdomyolysis/myopathy.” All candidate terms were clinically reviewed, and terms representing secondary complications, nonspecific manifestations, or events not directly attributable to skeletal muscle injury, such as renal-related events, were excluded. Additional Preferred Terms considered clinically relevant to myotoxicity but not included in the original SMQ were added. The final list of Preferred Terms used for case identification is presented in Supplementary Table S1.

### 2.2 Statistical analysis

Disproportionality analysis is a widely used statistical approach in pharmacovigilance for detecting potential safety signals based on disproportionate reporting of drug–event combinations (Bate and Evans, 2009). In this study, a case/non-case method was used to assess the reporting association between immune checkpoint inhibitors and myotoxicity in FAERS reports involving patients with lung cancer. Signal detection was performed using four complementary disproportionality measures, two frequentist and two Bayesian approaches were used for disproportionality analysis (Noguchi et al., 2021; Fusaroli et al., 2024). The frequentist approaches were quantified using the reporting odds ratio (ROR) and proportional reporting ratio (PRR). The Bayesian approaches included the Bayesian confidence propagation neural network (BCPNN) and the Multi-item Gamma Poisson Shrinker (MGPS), which generated the information component (IC) and empirical Bayes geometric mean (EBGM), respectively. ROR and PRR, provide straightforward and interpretable estimates of reporting disproportionality by comparing the relative reporting of a specific adverse event between the target drug and all other drugs (Rothman et al., 2004; Bate and Evans, 2009; Park et al., 2020). The Bayesian approaches incorporate shrinkage to improve the stability of estimates, particularly for drug–event combinations with small report counts (Liu et al., 2023). Signal detection thresholds were a lower 95% CI limit for ROR >1 with a ≥3, PRR ≥2 with χ^2^ ≥ 4, IC025 > 0 for BCPNN, and EBGM05 > 2 for MGPS. All signal detection algorithms were performed based on the standard 2 × 2 contingency table. The specific formula for the disproportionate analysis method can be seen in online Supplementary Table S2. Pearson chi-square test was used to compare the severity of subtypes.

## 3 Results

### 3.1 Descriptive analysis

The study period extended from the first quarter of 2015 to the fourth quarter of 2024, and the analysis focused on ICI-associated myotoxicity reported in patients with lung cancer. Based on the FAERS database, a total of 16,144,939 AE reports were retrieved. After screening and eligibility assessment, 45,266 ICI-associated myotoxicity reports were identified, of which 1,515 reports with ICIs as the primary suspect (PS) drug were included in the final analysis. The data processing flow is shown in Supplementary Figure S1. As shown in Figure 1A, the most frequently reported drug for ICI-related myotoxicity is Pembrolizumab (3.7%, n = 552). Figure 1B compares the proportion of adverse-event reports involving myotoxicity across the four ICIs. Myotoxicity represented a relatively small proportion of reports for all four agents, ranging from 2.38% to 3.70%, with the highest proportion observed for pembrolizumab and the lowest for durvalumab. We subsequently analyzed the clinical characteristics of the 1,515 lung cancer patients who developed myotoxicity. Among these 1,515 ICI-induced myotoxicity cases, a higher proportion of reports were from male patients (n = 904, 59.7%) compared to female patients (n = 508, 33.5%). The median age of the patients was 69.4 years (IQR 49.9–77.6), and most of the patients were between 65 and 85 years old (46.2%). Among the 592 cases with available weight data, 495 individuals had a weight between 50 and 100 kg. The largest number of reported cases came from medical doctors (n = 708, 46.7%), followed by consumers (n = 412, 27.2%). In addition, among the cases with AE outcomes, hospitalization (n = 534,33.9%) was the most frequently reported outcome. Among the cases with AE outcomes, hospitalization (n = 534,33.9%) was the most frequently reported outcome. Notably, although ICI-associated myotoxicity accounted for a relatively small proportion of reports compared with other common irAEs, fatal outcomes were documented in 20.1% of myotoxicity reports overall, and the corresponding proportion exceeded 20% for both nivolumab and pembrolizumab. Geographically, the AE reports for the four ICIs were predominantly from the United States (30.9%) and Japan (29.1%). More detailed information can be found in Table 1.

**FIGURE 1:**
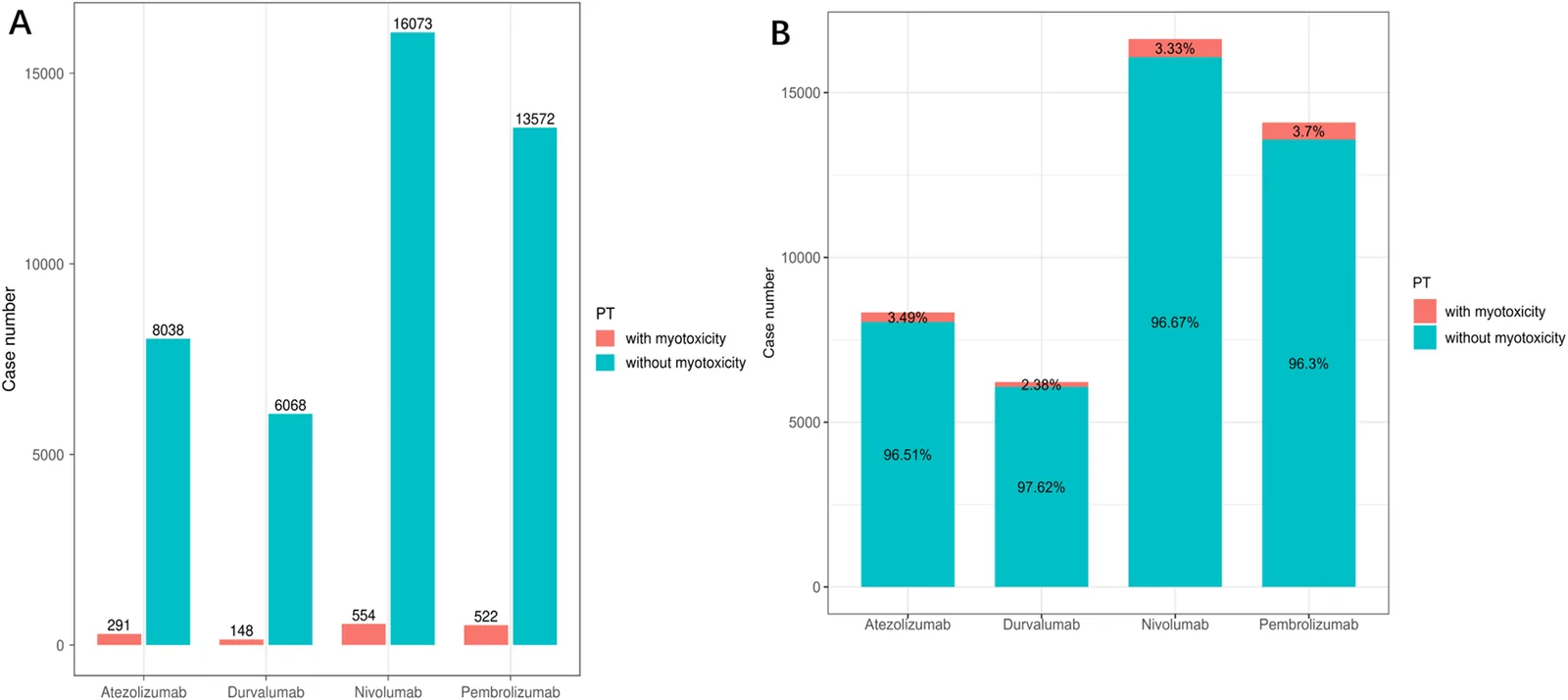
**(A)** The bar chart shows the number of four types of ICI-related myotoxic adverse events and the number of non-myotoxic events reported by lung cancer patients in the FAERS database from 2015 Q1 to 2024 Q4. **(B)** The proportional bar chart below shows the percentage of different ICI-related myotoxic events and non-myotoxic events in the FAERS database from 2015 Q1 to 2024 Q4.

**TABLE 1:** Characteristics of ICI-related myotoxicity.

Characteristics	Case number, n	Case proportion, %
​	1,515	​
Gender		
Female	508	33.5
Male	904	59.7
Unknown	103	6.8
Weight		
<50 kg	66	4.4
50–100 kg	495	32.7
>100 kg	31	2
Unknown	923	60.9
Age		
18–64	408	26.9
65–85	707	46.7
>85	25	1.7
Unknown	375	24.8
Reporters		
Consumer	412	27.2
Health professional	1,080	71.3
Unknown	23	1.5
Outcomes		
Death or life-threatening	389	25.7
Disability	29	1.9
Hospitalization	514	33.9
Other serious	373	24.6
Missing	210	13.9
Reported countries (top 5)		
United States	468	30.9
Japan	441	29.1
France	112	7.4
Italy	58	3.8
United Kingdom	56	3.7

N, number of cases with ICI-related myotoxicity.

The annual distribution of ICI-related myotoxicity reports is shown in Figure 2. Reporting frequency rose steadily from 2015 (n = 41), reaching a maximum of 222 cases in 2019. This peak was followed by a decline to 183 cases in 2020 and 155 cases in 2021. From 2022 to 2024, the annual report volume remained relatively stable, fluctuating between 133 and 156 cases.

**FIGURE 2:**
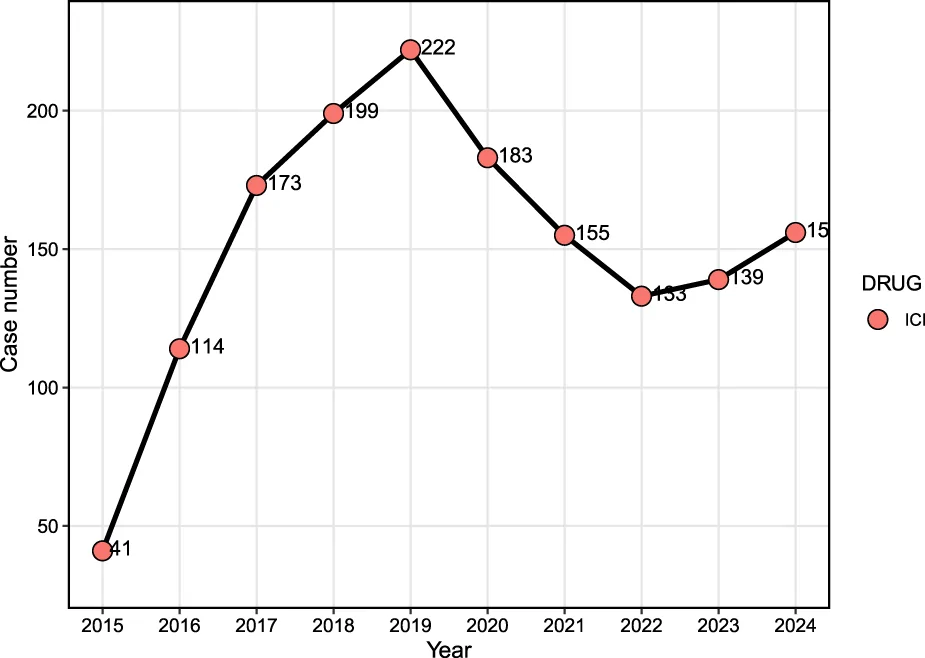
Annual distribution of ICI-related myotoxicity reports from 2015 to 2024.

### 3.2 Distribution of AEs at the PT level

We integrated the specific signal values of all four immune suppressants that induce myotoxicity in lung cancer patients. Table 2 presents the disproportionality signals for ICI-associated myotoxicity across the four agents, with signal strength varying among them. Specifically, nivolumab (ROR 1.20,95%CI:1.11–1.31), pembrolizumab (ROR 1.28,95%CI:1.17–1.39) and atezolizumab (ROR 1.25,95%CI:1.12–1.40) showed significant positive signals, with pembrolizumab showing the strongest association with muscle toxicity among these agents. In contrast, Durvalumab (ROR 0.94,95%CI:0.81–1.10) did not show a significant signal, suggesting that its muscle toxicity may be relatively stable.

**TABLE 2:** Safety adverse events among different ICI.

Drug	ICI-associated AEs n	ICI-associated myotoxicity n	ICI-associated	ROR(95%CI)	PRR(c^2^)	EBGM(EBM05)	IC(IC025)
			Myotoxicity PSn				
Nivolumab*	44,738	644	554	1.20(1.11–1.31)	1.20(19.23)	1.18 (1.10)	0.24(0.12)
Pembrolizumab*	40,329	613	522	1.28(1.17–1.39)	1.27(32.28)	1.24 (1.16)	0.31(0.19)
Atezolizumab*	21,865	329	291	1.25(1.12–1.40)	1.24 (15.10)	1.23 (1.12)	0.30(0.14)
Durvalumab	14,359	166	148	0.94(0.81–1.10)	0.94 (0.55)	0.95 (0.83)	−0.08(-0.31)

^*^ the number of reports; PS, primary suspect; ROR, reporting odds ratio; PRR, proportional reporting ratio; χ2, chi-squared; IC, information component; IC025, the lower limit of 95% CI, of the IC; EBGM, empirical Bayesian geometric mean; EBGM05, the lower limit of 95% CI, of EBGM.

Notably, eight positive disproportionality signals were identified, as presented in Table 3. Base on the number of reports, myalgia (ROR 1.25, 95%CI:1.1–1.41) was the most frequently reported AE, followed by muscular weakness (ROR 1.53, 95%CI:1.33–1.7), myositis (ROR 4.66, 95%CI:3.84–5.65) and musculoskeletal pain (ROR 1.64, 95%CI:1.32–2.04). To further characterize the reported clinical outcomes of these myotoxic events, we calculated the proportion of reports with a fatal outcome for each event category, defined as the number of reports with death as an outcome divided by the total number of reports for that event (Figure 3). These proportions varied across the different types of ICI-associated myotoxicity. Myalgia and myasthenia showed comparatively lower proportions of fatal outcomes, at 10.93% and 19.56%, respectively. Nevertheless, the relatively large numbers of reports for these two events contributed substantially to the overall number of ICI-associated myotoxic adverse events. The proportions of reports with a fatal outcome were 28.8% for myositis and 28.6% for dermatomyositis, with higher proportions were observed for polymyositis (42.86%) and rhabdomyolysis (38.83%). These findings indicate that some reported myotoxicity phenotypes were accompanied by relatively high proportions of fatal outcomes.

**TABLE 3:** The signal values of the eight types of ICI related Myotoxicity.

Preferred term (PTs)	Report number	ROR(95%Cl)	PRR(X^2^)	EBGM(EBGM05)	IC(IC025)
Myalgia	366	1.25 (1.1–1.41)	1.25 (12.55)	1.17 (1.06)	0.23 (0.06)
Muscular weakness	317	1.53 (1.33–1.75)	1.53 (37.75)	1.34 (1.2)	0.43 (0.24)
Myositis	267	4.66 (3.84–5.65)	4.65 (293.28)	2.4 (2.04)	1.26 (1.04)
Musculoskeletal pain	129	1.64 (1.32–2.04)	1.64 (20.66)	1.41 (1.18)	0.5 (0.2)
Rhabdomyolysis	103	1.56 (1.23–1.98)	1.56 (13.39)	1.36 (1.11)	0.45 (0.12)
Dermatomyositis	56	7.36 (4.49–12.05)	7.35 (86.74)	2.79 (1.85)	1.48 (0.98)
Myopathy	52	2.5 (1.73–3.63)	2.5 (25.17)	1.81 (1.32)	0.85 (0.37)
Polymyositis	28	8.99 (4.24–19.05)	8.99 (48.36)	2.94 (1.57)	1.56 (0.85)

PTs, preferred terms; ROR, reporting odds ratio; PRR, proportional reporting ratio; χ^2^: chi-squared; IC: information component; IC025: the lower limit of 95% CI, of the IC; EBGM, empirical Bayesian geometric mean; EBGM05, the lower limit of 95% CI, of EBGM.

**FIGURE 3:**
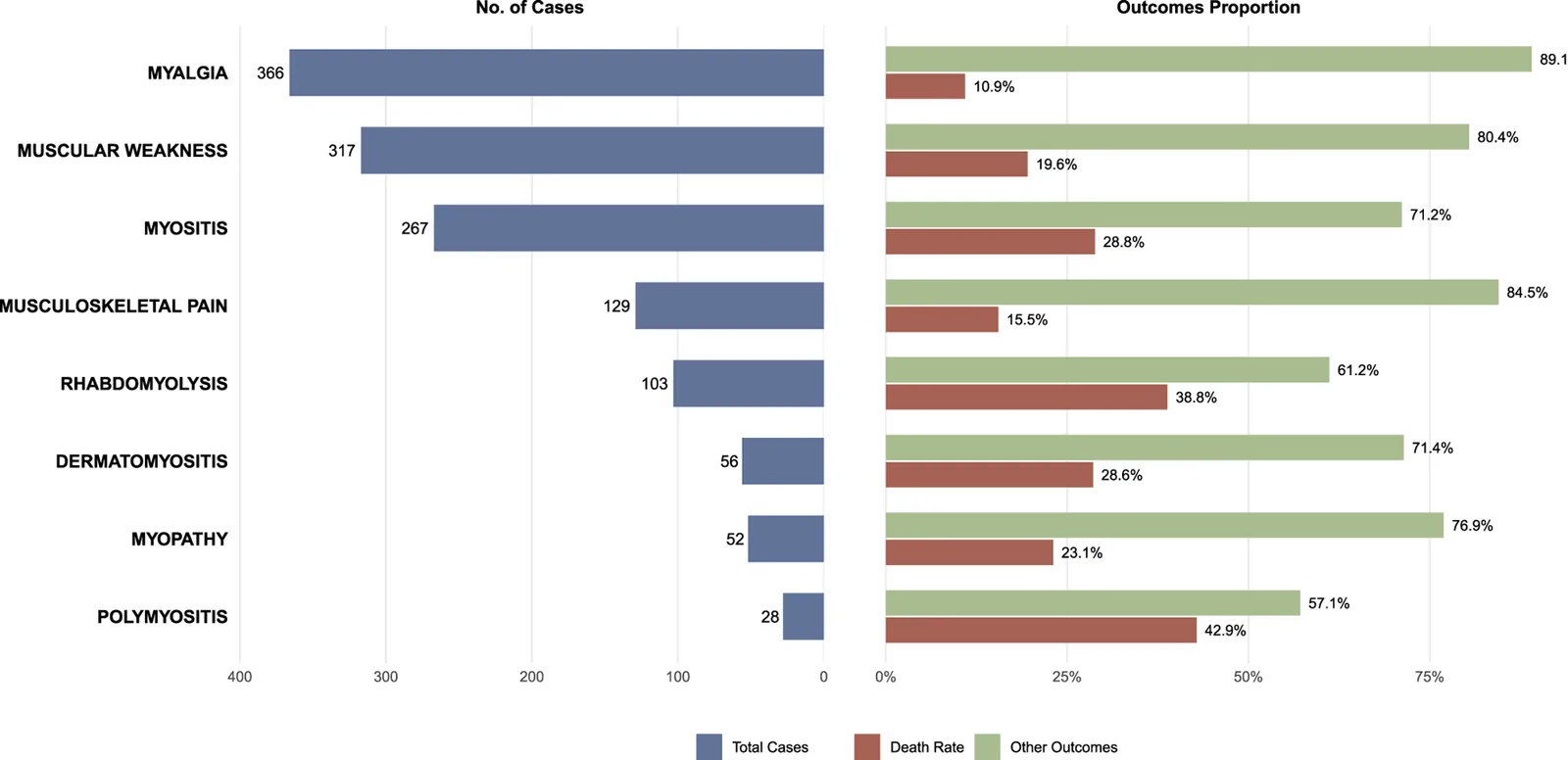
Case counts and fatal-outcome reporting of the eight significant myotoxic adverse events associated with ICIs.

### 3.3 Overlap toxicity and comparison between serious and non-serious groups for ICI-related myotoxicity

Among the 267 reports of myositis, four clinical phenotypes were identified. As shown in Figure 4A, myositis–myasthenia (MG) gravis overlap accounted for 14.6% of reports, whereas myositis–myocarditis (MC) overlap accounted for 18.0%. The distribution of implicated immune checkpoint inhibitors did not differ significantly across the four phenotype groups (Table 4, P = 0.868). In contrast, the distribution of reported clinical outcomes differed significantly among the phenotype groups (Table 4, P < 0.001). The proportion of reports with death as an outcome increased descriptively with the number of involved organs, ranging from 20.0% in isolated myositis to 46.7% in the Triple M phenotype. Reports involving Myositis + MC group and the Triple M phenotype had higher proportions of death or life-threatening outcomes, indicating that these overlap phenotypes were associated with less favorable reported outcomes. Figure 4B presents the distribution of reported outcomes across the four phenotypes. Detailed clinical characteristics of the included reports are provided in Supplementary Table S4.

**FIGURE 4:**
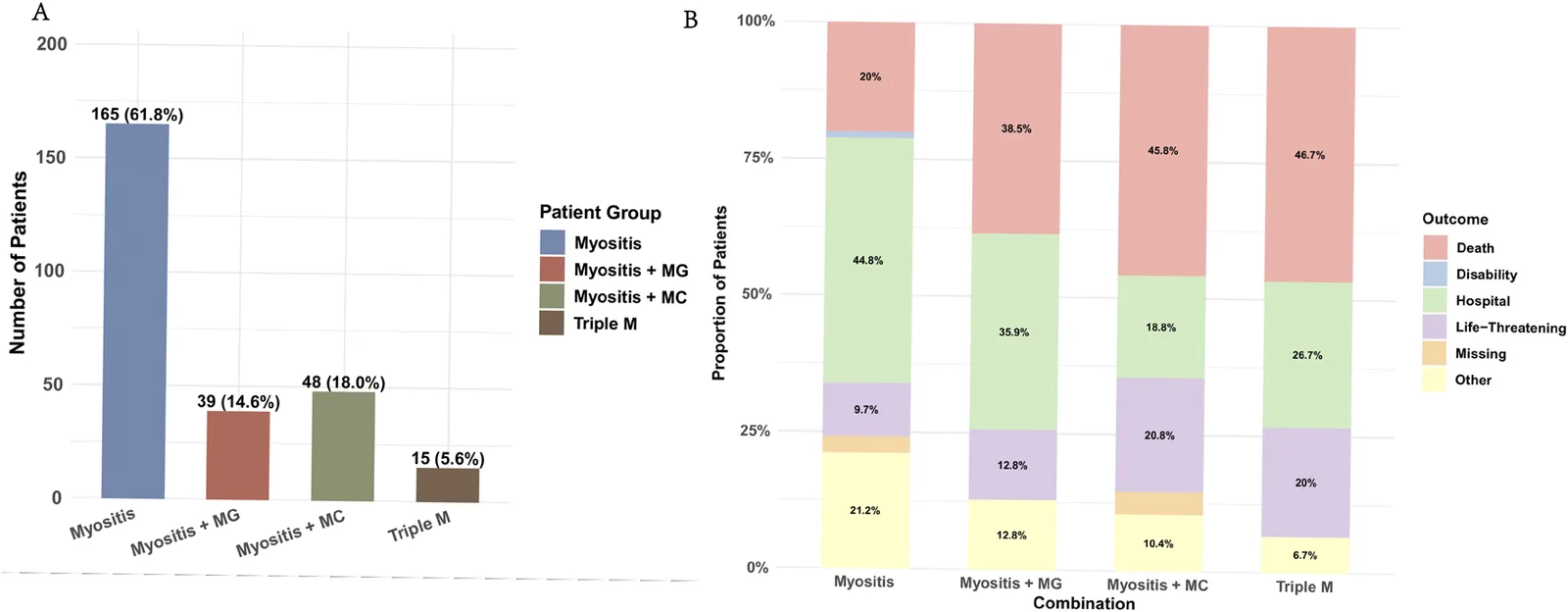
Patient distribution and clinical outcomes across patient groups in the FAERS cohort. **(A)** Number of patients in each clinical subgroup: myositis (n = 165, 61.8%), myositis + MG (n = 39, 14.6%), myositis + MC (n = 48, 18.0%), and Triple M (n = 15, 5.6%). **(B)** Proportions of clinical outcomes by patient group, including death, disability, hospitalization, life-threatening.

**TABLE 4:** Clinical characteristics and outcomes of patients with ICI-related myositis across four subphenotypes.

Clinical characteristics	Myositis (n = 165)	Myositis + MG (n = 39)	Myositis + MC (n = 48)	Triple M (n = 15)	P
**Drug, n (%)**	​	​	​	​	**0.607**
Nivolumab	59 (35.8)	13 (33.3)	16 (33.3)	5 (33.3)	​
Pembrolizumab	56 (33.9)	11 (28.2)	21 (43.8)	6 (40.0)	​
Atezolizumab	34 (20.6)	11 (28.2)	6 (12.5)	3 (20.0)	​
Durvalumab	16 (9.7)	4 (10.3)	5 (10.4)	1 (6.7)	​
**Outcome, n (%)**	​	​	​	​	**< 0.001** *
Death	33 (20.0)	15 (38.5)	22 (45.8)	7 (46.7)	​
Life-Threatening	16 (9.7)	5 (12.8)	10 (20.8)	3 (20.0)	​
Hospitalization	74 (44.8)	14 (35.9)	9 (18.8)	4 (26.7)	​
Other/Missing	42 (25.5)	5 (12.8)	7 (14.6)	1 (6.7)	​

ICI, immune checkpoint inhibitor; MG, myasthenia gravis; MC, myocarditis; Triple M, overlapping myositis, myasthenia gravis, and myocarditis; P < 0.05 was considered statistically significant.

### 3.4 Time to onset of ICI-associated myotoxicity

Time-to-onset data were calculable for 611 reports. The median TTO was 31 days [interquartile range (IQR), 14–82 days]. As shown in Figure 5A, 303 reports (49.6%) documented event onset within the first 30 days after treatment initiation, and a further 117 reports (19.1%) documented onset between 31 and 60 days. In addition, 11.1% of the AEs were reported to occur more than 6 months after treatment initiation. Figure 5B presents the distribution of TTO according to the implicated ICI. Atezolizumab had the numerically shortest median TTO at 21 days (IQR, 11–85 days), followed by nivolumab at 33 days (IQR, 14–74 days), pembrolizumab at 34 days (IQR, 15–105 days), and durvalumab at 36 days (IQR, 18–54.5 days). Although reported onset was concentrated in the early treatment period, reports with substantially longer TTO values were also identified. The longest recorded TTO was 1,652 days in a report involving nivolumab. TTO also varied across the predefined myotoxicity categories, with a statistically significant difference in the overall distributions among groups (Kruskal–Wallis test, P = 0.003; Figure 6). The musculoskeletal pain category had a relatively short median TTO of 28 days (IQR, 11–74 days), whereas the myopathy category had a longer median TTO of 52 days (IQR, 31–81 days). The median TTOs of the remaining myotoxicity categories ranged from 28.0 to 45.5 days.

**FIGURE 5:**
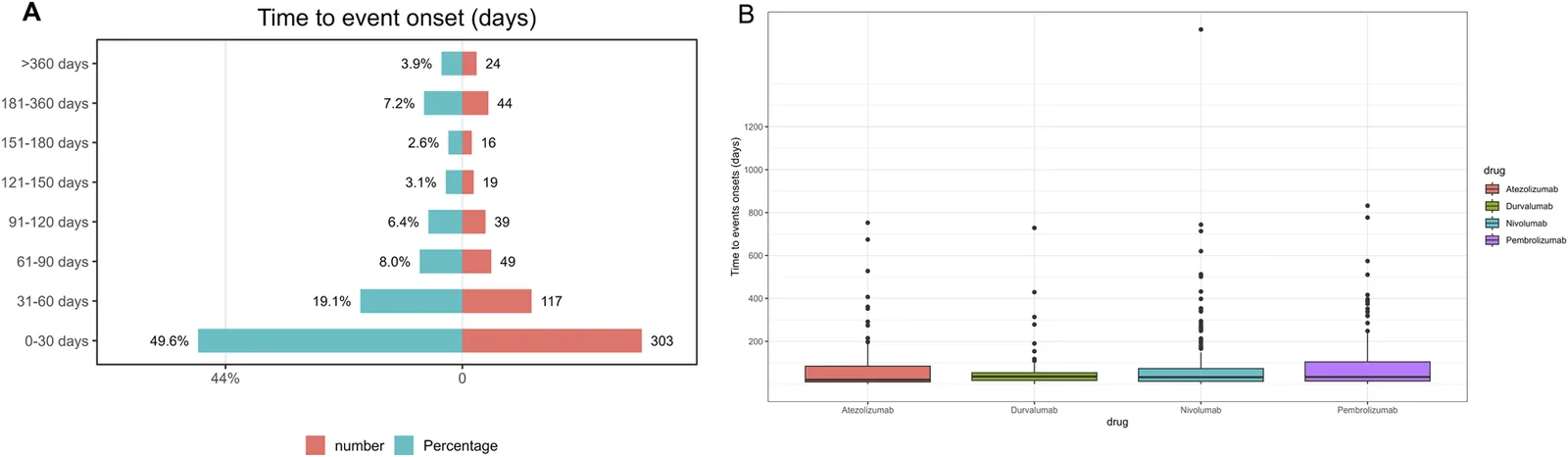
Time to onset analysis. **(A)** Overall time-to-onset profile of myotoxicity across four ICI agents. **(B)** Time to onset of four ICI-related myotoxicity.

**FIGURE 6:**
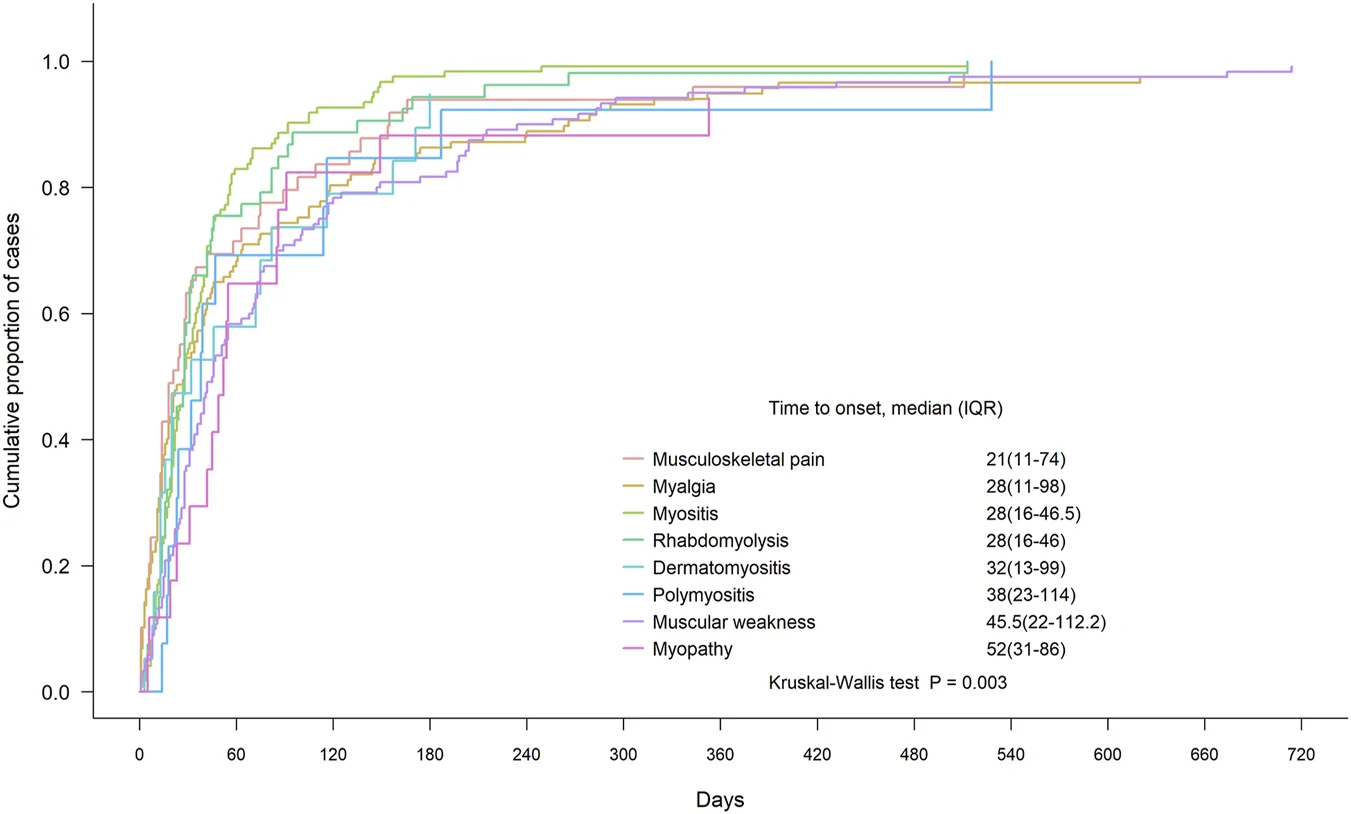
The cumulative incidence of different muscle toxicity associated with ICI.

## 4 Discussion

In this study, we used reports submitted to the FAERS database from the first quarter of 2015 through the fourth quarter of 2024 to systematically characterize muscle-related adverse events associated with immune checkpoint inhibitors in patients with lung cancer, thereby providing preliminary pharmacovigilance evidence on the reporting profile of ICI-associated myotoxicity. We compared the reporting patterns and disproportionality signals of muscle-related adverse events associated with four ICIs in lung cancer reports, characterized the clinical features of these events, and evaluated their time to onset. These findings provide pharmacovigilance evidence that may support clinical monitoring and treatment decision-making. We further assessed the reported proportion of triple-M overlap syndrome and the frequency of fatal outcomes across different overlap phenotypes, identifying significant differences in reported outcomes among these phenotypes. Collectively, our findings underscore the importance of early recognition and close monitoring of ICI-associated myotoxicity in patients with lung cancer, particularly severe multiorgan overlap syndromes such as triple-M syndrome.

Through signal detection analysis, nivolumab, pembrolizumab, and atezolizumab showed positive disproportionality signals for myotoxicity, myotoxicity-related events are not accidental in clinical practice (Peverelli et al., 2020; Hamada et al., 2021). Mechanistically, ICIs can disrupt immune homeostasis by enhancing the activation of T cells, natural killer cells and other immune effector cells, thereby triggering muscle tissue inflammation and injury (Matsubara et al., 2019; Matas-García et al., 2020; Liu et al., 2021). Previous studies have suggested that the blocking of the PD-1/PD-L1 pathway may lead to excessive activation of CD8 + T cells, and by destroying the regulation of the immune system, immune cells penetrate into muscle tissue, causing inflammation and muscle fiber damage (Läubli et al., 2018; Rajak, 2025). Clinically, it often manifests as myositis, polymyositis or other phenotypes. ICI-related toxicity is not limited to single organ involvement, and may evolve into multiorgan overlap syndrome, which may become the focus and difficulty in future immunotoxicity management.

After rigorous review, approximately 3.34% of the 45,266 initially screened ICI-related reports met the final inclusion criteria with myotoxicity as the primary suspect event. Although this proportion cannot be interpreted as clinical incidence, it expands our understanding of the spectrum of ICI- associated immune abnormalities and emphasizes the diversity of reported clinical manifestations. The analysis showed that male patients accounted for a larger proportion of reports, and the affected patients was mainly between 65 and 85 years old, with a median age of 69.4 years (IQR 49.9–77.6). The observed fatal-outcome reporting proportion of 20.1% is comparable to the findings of Paul Gougis et al. (Gougis et al., 2024), further highlighting the potential clinical severity of ICI-associated myotoxicity (Beecher et al., 2024).

In our disproportionality analysis, eight PTs met the predefined signal criteria, with myalgia and muscular weakness being the most frequently reported events. These symptoms may represent early or nonspecific manifestations of ICI-associated myotoxicity and can impair physical function, daily activities, social participation, and quality of life (Mamarabadi et al., 2025; Segal et al., 2025). However, myalgia and muscular weakness may be misattributed to cancer-related fatigue or the underlying disease, potentially delaying clinical evaluation and recognition of myotoxicity (Stroes et al., 2015; Tan and Song, 2025). This may result in potentially worsening muscle damage and the symptoms of the primary disease. In contrast, polymyositis and rhabdomyolysis were reported less frequently but were accompanied by higher proportions of fatal outcomes (Huerta-Alardín et al., 2005; Mendoza-Pinto et al., 2025). In the present analysis, fatal outcomes were recorded in 42.86% of polymyositis reports and 38.83% of rhabdomyolysis reports, whereas the corresponding proportions for several other myotoxic events were approximately 28%–34%. These findings suggest heterogeneity in the reported clinical outcomes of different myotoxicity phenotypes. These differences in outcomes may be attributed to specific clinical characteristics, involving multiple organ systems, particularly the heart, lungs, and kidneys (Peregud-Pogorzelska et al., 2018; Garg et al., 2020; Xia et al., 2022; Trybuch and Tarnacka, 2023), leading to fatal cascades of physiological dysfunction and severe metabolic disturbances, thereby narrowing the therapeutic window.

It is noteworthy that extra-muscular organs involvement in this condition is not an isolated event. Several studies have shown that about half of ICI-induced myositis often overlaps with other irAEs, especially with myocarditis (Jayan et al., 2024). In addition, recent meta-analyses and case reports indicate that some patients may present with a triple overlap of myositis, myocarditis, and myasthenia gravis, known as the Triple M Overlap Syndrome (Pathak et al., 2021; Raschi et al., 2023). Although this syndrome is relatively rare, it is one of the most clinically concerning phenotypes in the ICI toxicity spectrum. In the present study, death was reported in 46.7% of Triple M Overlap Syndrome, consistent with the 40%–60% mortality range reported in previous reviews and meta-analyses (Pathak et al., 2021; Sánchez-Camacho et al., 2025). Most often attributed to fatal arrhythmias, heart failure following myocardial involvement, and respiratory failure. Our results showed that the fatal-outcome reporting proportion of myositis overlapping myocarditis (45.8%) was similar to that of Triple M Overlap Syndrome (46.7%), suggesting that patients with myocarditis may have a higher clinical risk. However, because only 15 Triple M reports were identified, the precision and statistical power of this estimate are limited. Researchers have identified identical α-myosin-specific TCR clones in three ICI-related myocarditis patients, and these clones were present in both affected heart and skeletal muscles (Axelrod et al., 2022). This suggests that the overlapping symptoms may be related to the expansion of immune responses and the sharing of antigen expression (Levine, 2006; Beecher et al., 2024). In summary, ICI-related myotoxicity should not be viewed as isolated muscle damage but rather as a potential systemic immune pathological process involving interactions between key organs. Further research is needed to reveal the underlying mechanisms of ICI-induced myotoxicity and provide theoretical foundations for potential treatment targets.

Our results showed a significant difference in the reported time to onset of ICI-related myotoxicity (P = 0.003), which provided important clues for the early recognition and monitoring of immune-related adverse reactions in clinical practice. Specifically, TTO analysis showed that most of the muscle toxicity occurred within 1–2 months after ICI use (68.7%) (Sundarrajan et al., 2023), while a smaller proportion(3.9%) occurred after more than 1 year of treatment. Early symptoms such as musculoskeletal pain and myalgia often lack obvious specificity and may be misdiagnosed as other common adverse reactions or treatment side effects. Unlike early symptoms, late symptoms such as muscle weakness and myopathy may occur over a longer period after treatment, and these symptoms are usually associated with persistent activation of the immune system and immune-mediated chronic injury (Ma et al., 2021). These differences in onset time highlight the complexity of immune-mediated toxicity, Clinically, attention should be paid to early non-specific muscle symptoms, and long-term follow-up and monitoring of late symptoms should be strengthened.

Given the high mortality and overlapping syndromes of ICI-related myotoxicity, management should be multidisciplinary. We should establish a systematic monitoring protocol. This includes baseline assessments of muscle function, serum creatine kinase, and cardiac biomarkers prior to ICI initiation, followed by regular surveillance during treatment. For patients with overlapping toxicities, especially with systemic symptoms, cardiac function monitoring and support should be intensified (Wang et al., 2024). At the same time, attention should also be paid to the drug management of non-neoplastic drugs such as anti-dyslipidemia drugs, receptor blockers, antipsychotic drugs, donepezil and colchicine-induced myotoxicity. If there is no improvement after drug withdrawal, the treatment should be considered to terminate.

Beyond surveillance and risk stratification, exercise is a safe and effective adjunct for patients with myopathy after acute inflammation is controlled. Studies show that muscle strength and mass are independent survival predictors in lung cancer (Shiroyama et al., 2019). Good muscle health improves prognosis and may delay myotoxicity development (Mallet et al., 2021; Li Q. et al., 2024; Bettariga et al., 2025). In patients at high risk for sever AEs or overlapping syndrome at risk of severe progression, activity restriction is crucial until the inflammatory reaction resolve, helping to avoid worsening myotoxicity or rhabdomyolysis (Siciliano et al., 2019; Scott et al., 2021; Momma et al., 2022). If needed, muscle biopsy and immunosuppressive therapy (such as corticosteroids or intravenous immunoglobulin) should be considered.

Several limitations of this study warrant consideration. Firstly, as FAERS is a spontaneous reporting system, it is inherently subject to reporting bias, incomplete documentation, and a lack of denominator data for the total exposure population, true incidence, absolute mortality, and causal risk cannot be estimated (Guzel et al., 2026; Mutis Alan et al., 2026). Second, key clinical variables, including cancer histology and stage, performance status, and baseline autoimmune disease were unavailable or incompletely reported in FAERS. Consequently, residual confounding cannot be excluded, and the observed signals should not be interpreted as direct estimates of drug-specific risk or comparative safety. In addition, serious and fatal outcomes are recorded at the report level and may reflect the underlying malignancy, overall clinical condition, coexisting toxicities, or other treatments rather than being directly attributable to myotoxicity or a specific overlap syndrome. Therefore, differences in reported outcome proportions should be interpreted with caution. Triple M overlap syndrome was identified in a small subset of myositis-related reports, and the limited number of cases prevents definitive conclusions regarding its clinical significance. The proportions of reports with fatal outcome should not be interpreted as true clinical mortality rates. These findings should be considered hypothesis-generating and require further validation in prospective clinical studies. In clinical practice, the emergence of such myotoxic symptoms during ICI therapy necessitates rigorous monitoring, prompt risk assessment, and multidisciplinary management. Early identification and intervention remain pivotal in mitigating severe consequences and optimizing patient outcomes.

## 5 Conclusion

In general, ICI-related Myotoxicity is often covered up due to mild symptoms or the patient’s underlying cancer condition, leading to delayed recognition and intervention. Our study highlights the need to pay more attention to Myotoxicity during ICI treatment. Especially for high-risk and overlapping patients, timely monitoring and early recognition of muscle toxicity may support clinical management and improve treatment tolerance and quality of life. Our research will expand our understanding of the safe use of ICI and contribute important insights into the development of personalized cancer treatment strategies. Because FAERS is a spontaneous reporting system, the observed disproportionality patterns and outcome proportions should not be interpreted as estimates of incidence or evidence of causality. Further prospective studies and mechanistic investigations are needed to validate these findings, identify clinically useful predictors, and optimize monitoring and management strategies for ICI-associated myotoxicity.

## Data Availability

Publicly available datasets were analyzed in this study. This data can be found here: The datasets analyzed in this study are publicly available from the FDA Adverse Event Reporting System (FAERS) Quarterly Data Extract Files, U.S. Food and Drug Administration. The data can be accessed at: https://fis.fda.gov/extensions/FPD-QDE-FAERS/FPD-QDE-FAERS.html. No accession number is applicable.
