# Supplementary material for: Immune checkpoint inhibitor-related myotoxicity in lung cancer: a decade-long real-world pharmacovigilance analysis of the FAERS database
Source: Front Pharmacol. 2026 Sep 22;17:1875873. doi: 10.3389/fphar.2026.1875873 (PMC13638541; doi:10.3389/fphar.2026.1875873)
Supplement: Supplementary file 1 [file Supplementaryfile1.docx]

Supplementary Material

# 1 Supplementary Figures and Tables

For more information on Supplementary Material and for details on the different file types accepted, please see [here](https://www.frontiersin.org/guidelines/author-guidelines#supplementary-material).

## 1.1 Supplementary Figures

**Supplementary Figure 1.** **The flow chart of data processing** 2015Q1 refers to the data for the third quarter of 2015, 2024Q4 refers to the data for the third quarter of 2024.

**Supplementary Figure 2.** The number of reported cases of the first twenty types of ICI-related myotoxicity under different ICI treatment strategies. PT, preferred term.

## 1.2 Supplementary Tables

**Table 1. Standardized Medical Dictionary for Regulatory Activities (MedDRA) terms used for case identification.**

| **Serial Number** | **Myotoxicity Terminology** |
| --- | --- |
| 1 | Abnormal Emg |
| 2 | Achilles Tendon Discomfort |
| 3 | Acute Myopathy |
| 4 | Amyotrophy |
| 5 | Anal Sphincter Electromyography Abnormal |
| 6 | Antisynthetase Syndrome |
| 7 | Apocamnosis |
| 8 | Arthromyalgia |
| 9 | Axial Myopathy |
| 10 | Back Discomfort |
| 11 | Biopsy Muscle Abnormal |
| 12 | Blood Calcium Decreased |
| 13 | Blood Creatine Phosphokinase Abnormal |
| 14 | Blood Creatine Phosphokinase Abnormal Nos |
| 15 | Blood Creatine Phosphokinase Increased |
| 16 | Blood Creatine Phosphokinase Mm Increased |
| 17 | Blood Myoglobin Increased |
| 18 | Buttock Pain |
| 19 | Ck Increased |
| 20 | Compartment Syndrome |
| 21 | Cpk Increase |
| 22 | Cpk Increased |
| 23 | Cpk-Mm Increased |
| 24 | Creatine Kinase High |
| 25 | Creatine Kinase Increased |
| 26 | Creatine Phosphokinase Abnormal |
| 27 | Creatine Phosphokinase Increased |
| 28 | Creatine Phosphokinase Serum Increased |
| 29 | Dermatomyositis |
| 30 | Descending Muscle Weakness |
| 31 | Diabetic Myonecrosis |
| 32 | Diaphragm Muscle Weakness |
| 33 | Diastasis Of Muscle |
| 34 | Diastasis Recti Abdominis |
| 35 | Discomfort In Joints |
| 36 | Disorder Muscle |
| 37 | Dynapenia |
| 38 | Electromyogram Abnormal |
| 39 | Emg Abnormal |
| 40 | Exertional Rhabdomyolysis |
| 41 | Facial Myokymia |
| 42 | Fibromyalgia |
| 43 | Focal Myositis |
| 44 | Generalized Muscle Aches |
| 45 | Generalized Muscle Weakness |
| 46 | Granulomatous Myositis |
| 47 | Groin Discomfort |
| 48 | Haematoma Muscle |
| 49 | Haemorrhage Muscle |
| 50 | Hands Weakness Of |
| 51 | Hip Discomfort |
| 52 | Hypercreatinaemia |
| 53 | Hypocalcaemia |
| 54 | Hypothyroid Myopathy |
| 55 | Idiopathic Inflammatory Myopathy |
| 56 | Inclusion Body Myositis |
| 57 | Intercostal Myalgia |
| 58 | Interscapular Pain |
| 59 | Interstitial Myositis |
| 60 | Localized Muscle Pain |
| 61 | Localized Muscle Weakness |
| 62 | Lower Extremities Weakness Of |
| 63 | Lower Extremity Dysfunction |
| 64 | Lupus Myositis |
| 65 | Mitochondrial Myopathy Acquired |
| 66 | Muscle Ache |
| 67 | Muscle Atrophy |
| 68 | Muscle Biopsy Abnormal |
| 69 | Muscle Bleeding |
| 70 | Muscle Burning Sensation |
| 71 | Muscle Discomfort |
| 72 | Muscle Disorder |
| 73 | Muscle Disorder Nos |
| 74 | Muscle Dissolution |
| 75 | Muscle Enzyme Abnormal |
| 76 | Muscle Enzyme Increased |
| 77 | Muscle Fatigue |
| 78 | Muscle Fibrosis |
| 79 | Muscle Haemorrhage |
| 80 | Muscle Hypertrophy |
| 81 | Muscle Hypoxia |
| 82 | Muscle Inflammation |
| 83 | Muscle Mass |
| 84 | Muscle Necrosis |
| 85 | Muscle Oedema |
| 86 | Muscle Pain |
| 87 | Muscle Rigidity |
| 88 | Muscle Rupture |
| 89 | Muscle Soreness |
| 90 | Muscle Spasms |
| 91 | Muscle Strength Abnormal |
| 92 | Muscle Swelling |
| 93 | Muscle Tenderness Any Site |
| 94 | Muscle Tightness |
| 95 | Muscle Twitching |
| 96 | Muscle Weakness |
| 97 | Muscle Weakness Aggravated |
| 98 | Muscle Weakness Left-Sided |
| 99 | Muscle Weakness Lower Limb |
| 100 | Muscle Weakness Nos |
| 101 | Muscle Weakness Right-Sided |
| 102 | Muscle Weakness Trunk |
| 103 | Muscle Weakness Upper Limb |
| 104 | Muscular Contraction Wave Abnormal |
| 105 | Muscular Pain |
| 106 | Muscular Pains |
| 107 | Muscular Sarcoidosis |
| 108 | Muscular Weakness |
| 109 | Musculoskeletal Degeneration |
| 110 | Musculoskeletal Discomfort |
| 111 | Musculoskeletal Disorder |
| 112 | Musculoskeletal Disorder Nos |
| 113 | Musculoskeletal Pain |
| 114 | Musculoskeletal Toxicity |
| 115 | Myalgia |
| 116 | Myalgia Aggravated |
| 117 | Myalgia Intercostal |
| 118 | Myalgia Of Lower Extremities |
| 119 | Myalgia Upper Extremities |
| 120 | Myasthenia |
| 121 | Myofascial Pain Syndrome |
| 122 | Myofascial Spasm |
| 123 | Myoglobin Blood Increased |
| 124 | Myoglobin Blood Present |
| 125 | Myoglobin Urine Increased |
| 126 | Myoglobin Urine Present |
| 127 | Myoglobinaemia |
| 128 | Myoglobinemia |
| 129 | Myoglobinuria |
| 130 | Myokymia |
| 131 | Myonecrosis |
| 132 | Myopathy |
| 133 | Myopathy Aggravated |
| 134 | Myopathy Endocrine |
| 135 | Myopathy Steroid |
| 136 | Myopathy Steroid-Induced |
| 137 | Myopathy Toxic |
| 138 | Myopathy Unspecified |
| 139 | Myorrhexis |
| 140 | Myosclerosis |
| 141 | Myositis |
| 142 | Myositis-Like Syndrome |
| 143 | Myotendinal Junction Pain |
| 144 | Myotenositis |
| 145 | Neck Discomfort |
| 146 | Neck Muscle Weakness |
| 147 | Necrotising Myositis |
| 148 | Necrotizing Myositis |
| 149 | Neuromuscular Blockade |
| 150 | Neuromuscular Weakness |
| 151 | Neuropathic Muscular Atrophy |
| 152 | Nonspecific Abnormal Emg |
| 153 | Nuchal Rigidity |
| 154 | Omalgia |
| 155 | Orbital Myositis |
| 156 | Osteoarticular Pain |
| 157 | Other Non-Articular Rheumatism |
| 158 | Pain Muscle |
| 159 | Paradoxical Masseter Muscle Bulging |
| 160 | Phosphokinase Creatine Serum Increased |
| 161 | Plasma Creatine Phosphokinase Abnormal |
| 162 | Plasma Creatine Phosphokinase Increased |
| 163 | Polymyalgia |
| 164 | Polymyalgia Aggravated |
| 165 | Polymyalgia Worsened |
| 166 | Polymyositis |
| 167 | Proximal Muscle Weakness |
| 168 | Proximal Myopathy |
| 169 | Proximal Myopathy Aggravated |
| 170 | Rhabdomyolysis |
| 171 | Rhabdomyolysis-Induced Renal Failure |
| 172 | Rheumatics |
| 173 | Rheumatism |
| 174 | Rupture Of Muscle Non-Traumatic |
| 175 | Sarcopenia |
| 176 | Satoyoshi Syndrome |
| 177 | Scapula Pain |
| 178 | Serum Creatine Phosphokinase Abnormal |
| 179 | Serum Creatine Phosphokinase Increased |
| 180 | Shoulder Blade Pain |
| 181 | Shoulder Discomfort |
| 182 | Shoulder Pain |
| 183 | Shoulder Soreness |
| 184 | Steroid Myopathy |
| 185 | Symptomatic Inflammatory Myopathy |
| 186 | Tenderness Muscle |
| 187 | Tendon Discomfort |
| 188 | Thyrotoxic Myopathy |
| 189 | Tight Hamstring Syndrome |
| 190 | Torn Muscle |
| 191 | Torticollis |
| 192 | Toxic Myopathy |
| 193 | Traumatic Myositis Ossificans |
| 194 | Trigger Points |
| 195 | Trismus |
| 196 | Upper Extremity Dysfunction |
| 197 | Uraemic Myopathy |
| 198 | Urine Myoglobin Increased |
| 199 | Vertebrogenic Pain Syndrome |
| 200 | Weakness In Extremity |
| 201 | Weakness Muscle |
| 202 | Weakness Of Arms |
| 203 | Weakness Of Limbs |
| 204 | Weakness Of Upper Extremities |

**Table 2**.

**Four-cell table of proportional disequilibrium method**

| Drugs | Target adverse reaction | Other adverse reactions | Total |
| --- | --- | --- | --- |
| Target drug | a | b | a+b |
| Other drugs | c | d | c+d |
| Total | a+c | b+d | n=a+b+c+d |

a: the number of reports containing both the suspect drug and the suspect adverse drug reaction; b: the number of reports containing the suspect adverse drug reaction with other medications (except the drug of interest); c: the number of reports containing the suspect drug with other adverse drug reactions (except the event of interest); d: the number of reports containing other medications and other adverse drug reactions; n: the number of all reports.

**Four major algorithms used for signal detection.**

| Algorithms | Equation | Criteria |
| --- | --- | --- |
| ROR | ROR=ad/b/c | lower limit of 95% CI>1, N≥3 |
|  | 95%CI=e^ln(ROR)±1.96(1/a+1/b+1/c+1/d)^0.5^ |  |
| PRR | PRR=a(c+d)/c/(a+b) | PRR≥2, χ^2^≥4, N≥3 |
|  | χ^2^=[(ad-bc)^2](a+b+c+d)/[(a+b)(c+d)(a+c)(b+d)] |  |
| BCPNN | IC=log_2_a(a+b+c+d)(a+c)(a+b) | IC025>0 |
|  | 95%CI= E(IC) ± 2V(IC)^0.5 |  |
| MGPS | EBGM=a(a+b+c+d)/(a+c)/(a+b) | EBGM05>2 |
|  | 95%CI=e^ln(EBGM)±1.96(1/a+1/b+1/c+1/d)^0.5^ |  |

Abbreviation: a, number of reports containing both the target drug and target adverse drug reaction; b, number of reports containing other adverse drug reaction of the target drug; c, number of reports containing the target adverse drug reaction of other drugs; d, number of reports containing other drugs and other adverse drug reactions. 95%CI, 95% confidence interval; N, the number of reports; χ2, chi-squared; IC, information component; IC025, the lower limit of 95% CI of the IC; E(IC), the IC expectations; V(IC), the variance of IC; EBGM, empirical Bayesian geometric mean; EBGM05, the lower limit of 95% CI of EBGM.

**Table 3 Character of patients with ICI-related myotoxicity, stratified by subphenotype**

| Characteristics | ATEZOLIZUMAB | DURVALUMAB | NIVOLUMAB | PEMBROLIZUMAB | Total |
| --- | --- | --- | --- | --- | --- |
| SEX |  |  |  |  |  |
| Female | 15（27.78%） | 4（15.38%） | 25（26.88%） | 23（24.47%） | 67（25.09%） |
| Male | 20（37.04%） | 20（76.92%） | 63（67.74%） | 69（73.40%） | 172（64.42%） |
| Missing | 19（35.19%） | 2（7.69%） | 5（5.38%） | 2（2.13%） | 28（10.49%） |
| Weight |  |  |  |  |  |
| ＜50 kg | 8（14.81%） | 0（0.00%） | 1（1.08%） | 9（9.57%） | 18（6.74%） |
| ＞100 kg | 1（1.85%） | 1（3.85%） | 4（4.30%） | 4（4.26%） | 10（3.75%） |
| 50～100 kg | 13（24.07%） | 8（30.77%） | 32（34.41%） | 35（37.23%） | 88（32.96%） |
| Missing | 32（59.26%） | 17（65.38%） | 56（60.22%） | 46（48.94%） | 151（56.55%） |
| AGE |  |  |  |  |  |
| >85 | 2（3.70%） | 0（0.00%） | 2（2.15%） | 2（2.13%） | 6（2.25%） |
| 18～64.9 | 11（20.37%） | 2（7.69%） | 30（32.26%） | 22（23.40%） | 65（24.34%） |
| 65～85 | 15（27.78%） | 14（53.85%） | 49（52.69%） | 56（59.57%） | 134（50.19%） |
| Missing | 26（48.15%） | 10（38.46%） | 12（12.90%） | 14（14.89%） | 62（23.22%） |
| Reporters |  |  |  |  |  |
| Consumer | 1（1.85%） | 0（0.00%） | 14（15.05%） | 30（31.91%） | 45（16.85%） |
| Health-Professional | 16（29.63%） | 5（19.23%） | 9（9.68%） | 6（6.38%） | 36（13.48%） |
| Lawyer | 0（0.00%） | 0（0.00%） | 0（0.00%） | 0（0.00%） | 0（0.00%） |
| Physician | 35（64.81%） | 16（61.54%） | 52（55.91%） | 44（46.81%） | 147（55.06%） |
| Other Health-Professional | 0（0.00%） | 0（0.00%） | 14（15.05%） | 9（9.57%） | 23（8.61%） |
| Pharmacist | 2（3.70%） | 1（3.85%） | 4（4.30%） | 5（5.32%） | 12（4.49%） |
| Missing | 0（0.00%） | 4（15.38%） | 0（0.00%） | 0（0.00%） | 4（1.50%） |
| Outcomes |  |  |  |  |  |
| Congenital Anomaly | 0（0.00%） | 0（0.00%） | 0（0.00%） | 0（0.00%） | （0.00%） |
| Death | 13（24.07%） | 5（19.23%） | 29（31.18%） | 30（31.91%） | 77（28.84%） |
| Disability | 1（1.85%） | 0（0.00%） | 1（1.08%） | 0（0.00%） | 2（0.75%） |
| Hospitalization - Initial or Prolonged | 22（40.74%） | 10（38.46%） | 33（35.48%） | 36（38.30%） | 101（37.83%） |
| Life-Threatening | 1（1.85%） | 6（23.08%） | 12（12.90%） | 15（15.96%） | 34（12.73%） |
| missing | 3（5.56%） | 1（3.85%） | 1（1.08%） | 2（2.13%） | 7（2.62%） |
| Other Serious (Important Medical Event) | 14（25.93%） | 4（15.38%） | 17（18.28%） | 11（11.70%） | 46（17.23%） |
| REPORTER_COUNTRY |  |  |  |  |  |
| CHINA | 6（11.11%） | 0（0.00%） | 0（0.00%） | 1（1.06%） | 7（2.62%） |
| FRANCE | 2（3.70%） | 3（11.54%） | 14（15.05%） | 11（11.70%） | 30（11.24%） |
| ITALY | 2（3.70%） | 1（3.85%） | 3（3.23%） | 2（2.13%） | 8（3.00%） |
| JAPAN | 32（59.26%） | 9（34.62%） | 36（38.71%） | 34（36.17%） | 111（41.57%） |
| UNITED STATES | 3（5.56%） | 3（11.54%） | 12（12.90%） | 13（13.83%） | 31（11.61%） |
| Othere | 9（16.67%） | 10（38.46%） | 28（30.11%） | 33（35.11%） | 80（29.96%） |

**Table 4**: Comparison between the serious and non-serious groups for ICI-related myotoxicity

| **Type of PTs, n(%)** | **Serious case** | **Non_Serious case** | **P.value** | **χ2** |
| --- | --- | --- | --- | --- |
| Antisynthetase syndrome | 1(0.0027) | 1(0.00072) | 0.885 | 1.033 |
| Blood calcium decreased | 4(0.011) | 9(0.0065) | 0.587 | 0.784 |
| Blood creatine phosphokinase abnormal | 2(0.0055) | 4(0.0029) | 0.801 | 0.570 |
| Blood creatine phosphokinase increased | 30(0.082) | 113(0.081) | ＞0.999 | 0.002 |
| Dermatomyositis | 16(0.044) | 40(0.029) | 0.200 | 2.100 |
| Hypocalcaemia | 18(0.049) | 40(0.029) | 0.075 | 3.785 |
| Inclusion body myositis | 1(0.0027) | 3(0.0022) | ＞0.999 | 0.042 |
| Muscle atrophy | 5(0.014) | 11(0.0079) | 0.471 | 1.062 |
| Muscle disorder | 2(0.0055) | 4(0.0029) | 0.801 | 0.570 |
| Muscle rigidity | 2(0.0055) | 1(0.00072) | 0.213 | 3.828 |
| Muscle spasms | 6(0.016) | 78(0.056) | 0.002 | 10.027 |
| Muscle twitching | 2(0.0055) | 5(0.0036) | 0.969 | 0.255 |
| Muscular weakness | 62(0.17) | 255(0.18) | 0.588 | 0.381 |
| Musculoskeletal disorder | 5(0.014) | 12(0.0087) | 0.565 | 0.766 |
| Musculoskeletal pain | 20(0.055) | 109(0.079) | 0.151 | 2.398 |
| Myalgia | 40(0.11) | 326(0.24) | ＜0.001 | 27.517 |
| Myopathy | 12(0.033) | 40(0.029) | 0.817 | 0.164 |
| Myositis | 77(0.21) | 190(0.14) | 0.001 | 12.241 |
| Necrotising myositis | 3(0.0082) | 2(0.0014) | 0.108 | 4.664 |
| Polymyositis | 12(0.033) | 16(0.012) | 0.008 | 8.368 |
| Rhabdomyolysis | 40(0.11) | 63(0.045) | ＜0.001 | 21.502 |
| Trismus | 2(0.0055) | 4(0.0029) | 0.801 | 0.570 |

PTs, preferred terms; n, the number of reports.
